# Measuring change in domains of health-related quality of life and well-being in Black or African American adolescent football athletes from rural communities across a single football season

**DOI:** 10.1017/cts.2026.10794

**Published:** 2026-07-17

**Authors:** Jessica Wallace, Justin E. Karr, Danae Delfin Talley, Eric Ingram, Claire Brewer, Trevor Anesi, Mary Margaret Williamson, Zachary Y. Kerr, Tamaria Hibbler, Douglas Terry, Aaron Yengo-Kahn

**Affiliations:** 1 Health Science, The University of Alabama, Tuscaloosa, AL, USA; 2 University of Kentucky, Lexington, KY, USA; 3 University of South Florida, Tampa, FL, USA; 4 Vanderbilt University School of Medicine, Nashville, TN, USA; 5 University of New Mexico, Albuquerque, NM, USA; 6 The University of North Carolina at Chapel Hill, Chapel Hill, NC, USA; 7 University of Arkansas, Fayetteville, AR, USA; 8 Vanderbilt University Medical Center, Nashville, TN, USA; 9 Children’s Hospital of Orange County, Orange, CA, USA

**Keywords:** Athlete well-being, adolescent, football athletes, life satisfaction, health-related quality of life

## Abstract

**Background:**

Physical activity and team sport participation in adolescence are associated with positive health outcomes, stronger social skills, and improved mental health. However, negative health consequences are frequently associated with contact/collision sports like football. The objective of this study was to examine any change in health-related quality of life (HRQoL) and pediatric well-being among adolescent males after participating in a single season of football.

**Method:**

A cross-sectional study design was utilized in a high school football setting. Participants included Black/African American adolescent male football players (*n* = 83) from five schools within rural Alabama. Pediatric HRQoL and well-being measures were administered at preseason and postseason, including Life Satisfaction, Emotional Support, Psychological Stress, Physical Stress, Depressive Symptoms, Sleep Disturbance, and Peer Relationships. Based on normative data, participants’ scores were classified as within normal limits (WNL) (*T*-score = 40–60) or clinically reduced (*T*-score < 40) or elevated (*T*-score > 60) depending on orientation of each questionnaire. Each outcome from pre-to-postseason evaluation was compared using a within-persons *t*-test.

**Results:**

Most participants (69.9–84.3%) had HRQoL/well-being scores WNL and scores remained unchanged at postseason evaluations. However, on average, physical (*p* = 0.035, *d* = 0.24) and psychological stress (*p* = 0.014, *d* = 0.28) showed small, but significant increases from pre-to-postseason.

**Conclusions:**

Modest increases in physical and psychological stress were found from preseason to postseason. Both football and non-football-related stressors could have contributed to this change. Other HRQoL/well-being scores generally showed no change. Though the health risks associated with contact/collision sports deserve attention, current findings suggest a season of football may not be associated with adverse changes in HRQoL/well-being.

## Introduction

The term health-related quality of life (HRQoL) is multidimensional and encompasses a sense of subjective well-being [1]. The objective and subjective experiences of children and adolescents within their households and communities are an important factor in both short- and long-term health and development [2]. Evidence suggests that those who report positive well-being in adolescence are more likely to experience positive well-being in adulthood [3]. Subjective well-being may include overall life satisfaction, physical and behavioral health, psychological health, and social-emotional health.

The World Health Organization defines life satisfaction as a personal appraisal of overall fulfillment and the quality of one’s life, and it has received growing consideration as an indicator of global health among youth [4]. Adolescents with higher life satisfaction display greater academic and social functioning [5,6]. Both physical and psychological stress play a key role in overall well-being as well. Children and adolescents who endure severe or long-term physical stress are at elevated risk for poorer physical, cognitive, and social–emotional functioning [7–9]. Similar negative findings are also associated with sleep disturbances [7–9] and psychological stress experiences including anxiety and depression [10]. Because of the various impacts on overall health and well-being, it is imperative research targets a more comprehensive understanding of adolescent subjective and personal experiences [6], especially in rural communities where there is limited infrastructure to support health and well-being.

Fortunately, physical activity during adolescence is associated with positive health outcomes such as higher cardiorespiratory capacity, stronger muscles and bones, improved academic and cognitive performance, improved mental health, and developed social skills [11,12]. In particular, participation in organized sport has also been shown to elicit many health benefits for this age group. For example, former student-athletes report higher levels of self-esteem and happiness as adults than individuals who did not participate in sports [13]. Participation in team sports was also found to be associated with improved adult mental health in those exposed to adverse childhood experiences [14]. Further, emotional support and peer relationships have been identified as mediators of stress experiences in adolescent populations thus, participating in sport may offer psychological resources innate to the sports team experience. Exploring the role of sports participation in adolescent health is imperative, as physical activity has been shown to correlate with greater overall well-being [15,16].

Many adolescents get their physical activity from organized sport, with 56% of middle and high school students participating in team sports [17]. However, in 2018–2019, there was a decline in the number of adolescents participating in organized sport in the United States (US) [17]. This marked the first decline in organized sport participation in 30 years [17] and team sports participation took another notable hit during the start of the pandemic in 2020 and into 2021 [18]. Football saw the largest decrease in participation, potentially due to concerns over the short- and long-term consequences of injuries associated with participation in a contact/collision sport [19]. There were an estimated 2.6 million emergency department visits due to pediatric football injuries from 2010 to 2019, with 80,000 and 150,000 related to concussions among youth and high school football players, respectively [20]. Other common injuries include strains, sprains, fractures, contusions, and abrasions [20].

Despite risk of injury and recent decline in football participation, it remains the most popular sport in the US with around 5 million youth and adolescents participating annually [17,21]. According to the National Federation of State High School Associations, the southeastern US has some of the highest high school football participation rates in the country [21]. The southeastern US also has some of the highest percentages of rural populations [22]. Historically, rural adolescents are less likely to be physically active compared to their urban counterparts and may face additional barriers to organized sport participation such as higher concentrations of poverty, greater physical distances to travel, and a lack of reliable transportation [23].

This geographical region is also home to the largest Black and African American population in the US, with Alabama having the largest Black and African American population in the region [24]. Research has consistently found that Black and African Americans report disproportionately low HRQoL across the lifespan and have the lowest life expectancy compared to any other racial/ethnic group in the country [25]. HRQoL differs for minoritized populations due to pervasive educational, economic, social, and health inequities [25,26]. Most studies have focused on urban and suburban communities when exploring relationships between physical activity and various facets of health, and have largely neglected the unique needs of rural and minoritized populations, particularly within sport.

While most current literature on adolescent well-being involves general populations, this study has an intentional and novel focus on a sample of adolescent Black and African American athletes playing high school football from rural communities in Alabama. Though the role of sports in promoting general health and wellness is understood, no study has analyzed HRQoL measures both pre- and postseason to understand the effects of a single season of football participation. The purpose of this study was to examine any changes on measures of pediatric HRQoL and well-being across a season of high school football participation. Given the exploratory nature of the study, we adopted a null hypothesis that there would be no significant change from pre-to-postseason measures.

## Materials and method

### Participants

A sample of 126 American football players were recruited to participate from five high schools across four rural counties within Alabama. Participants were excluded from analyses if they had incomplete data on any outcome variable included in analysis (*n* = 43). There was limited missing data on measures at preseason evaluations (*n* range: 4 to 8), but more substantial missing data on measures at postseason evaluations (*n* range: 32–39). The final sample included 83 adolescent males (age: *M* = 15.6, SD = 1.1, range: 13–17; 100% Black or African American) with available pre-post data on all measures included in analysis. Further, in the final sample, 18.1% (*n* = 15) reported a history of concussion and 92.8% (*n* = 77) were eligible for free-or-reduced lunch. See Table 1 for complete health history and socio-demographic variables.

**Table 1.** Participant’s sociodemographic and health history characteristics Table 1 long description.

Health history characteristics	Included participants (*n* = 83)		Excluded participants (*n* = 43)	
	*n*	%	*n*	%
Age	*M* = 15.62	SD = 1.1	*M* = 15.76	SD = 1.4
Gender				
Male	83	100	42	97.7
Female	0	0	1	2.3
Race				
White	0	0	9	20.9
Black	83	100	31	72.1
Asian	0	0	1	2.3
Multiracial	0	0	2	4.7
Prior concussion	15	18.1	6	14.0
Number of prior concussions				
0/no response	68	81.9	37	86.0
1	12	14.5	5	11.6
2	3	3.6	1	2.3
Undiagnosed concussions	6	7.2	1	2.3
Skipped a year of school	0	0	0	0
Repeated a year of school	6	7.2	1	2.3
Previous history of:				
Headaches	60	72.3	32	74.4
Migraines	33	39.8	19	44.2
Motion sickness	8	9.6	11	25.6
Brain Injury/surgery	2	2.4	2	4.7
Seizure/epilepsy	1	1.2	2	4.7
Amblyopia/lazy eye	4	4.8	2	4.7
Vision problems	15	18.1	11	25.6
Anxiety	16	19.3	7	16.3
Depression	14	16.9	9	20.9
ADD/ADHD	11	13.3	5	11.6
Autism/asperger’s	1	1.2	1	2.3
Learning disorders	3	3.6	0	0
Eligible for free/reduced lunch	77	92.8	32	74.4

*Note:* Included participants describes those who had available data for preseason and postseason evaluations. Excluded participants describes those who did not have postseason data available.

### Measures

Participants were administered an electronic survey using scales with established validity in pediatric populations from the National Institutes of Health Toolbox Application (NIH Toolbox) and Patient-Reported Outcomes Measurement Information System (PROMIS). These pediatric scales encompass physical, mental, emotional and social domains of HRQoL and well-being. The survey consisted of self-report measures that assessed the subjective experience of HRQoL and well-being. General Life Satisfaction and overall Emotional Support were assessed using measures from the NIH Toolbox. The General Life Satisfaction measure contained 5 items graded on a 7-point Likert scale and assessed the cognitive evaluation of liking elements of one’s life or not. The Emotional Support measure contained 7 items graded on a 5-point Likert scale and questions measured participant’s perception and availability of individuals in one’s social network who listen, care, show empathy, and understanding. Higher scores indicated higher levels of perceived satisfaction with life and emotional support, both positive indicators of HRQoL.

Five other HRQoL indicators were assessed using PROMIS measures. These included Psychological Stress, Physical Stress, Depressive Symptoms, Sleep Disturbance, and Peer Relationships measures. Psychological Stress, Depressive Symptoms, and Peer Relationship measures all contained 8 items graded on a 5-point Likert scale. For all PROMIS measures, higher scores indicate more of that perceived construct. The Psychological Stress measure assesses participants’ stress experiences through perceptions of controllability, cognitive disruption, anger and fear [27]. Conversely, the Physical Stress scale assesses physical manifestations of stress such as gastrointestinal distress, disruptions to sleep, heart palpitations, physical pain, and agitation [28]. Therefore, higher scores on either stress measure are less desirable. The Depressive Symptoms measure focuses on negative perceptions of self and social connections, anhedonia, and other domains or depression [29]. The Sleep Disturbance measure assesses difficulty falling and staying asleep, as well as daytime functioning [30]. Higher scores in Depressive Symptoms and Sleep Disturbance could indicate worse functioning [29,30]. Lastly, the Peer Relationship measure assesses the quantity and quality of social support derived from peers, and as such, higher scores indicate better social functioning [31].

### Procedure

This study was approved by the institutional review board at The University of Alabama prior to the recruitment and consenting of participants and met the ethical human subjects research requirements. Parent consent was obtained for each participant. Following parent consent, informed assent was obtained, and participants were provided with details outlining the study. Participants completed the survey both pre- and postseason. Preseason data collection occurred within the first two weeks of preseason practice, and postseason data collection occurred within two weeks after the last competition was played. A quiet classroom space on the campus of each high school was identified for participants to complete the survey. Participants were given an iPad with the preloaded pediatric measures and read standardized instructions before beginning. Each participant was informed that they could skip survey items or withdraw from the study at any time. Completion of the survey took approximately 10–15 minutes. Participants were incentivized to complete both pre- and postseason surveys with gift cards.

### Statistical analysis

All outcome measures converted to norm-referenced *T*-scores (*M* = 50, SD = 10), which compare ratings for each scale to the general US population. Participants were compared on each outcome from preseason to postseason evaluations using a within-persons *t*-test. A finding of *p* < 0.05 indicated a significant change between evaluations. A Cohen’s *d* value was calculated for the pre-post comparison as an estimate of effect size, with rule-of-thumb qualitative descriptors used to interpret the magnitude of change on each measure (i.e., *d* = 0.20, small; *d* = 0.50, medium, *d* = 0.80, large). For each outcome measure, participants’ *T*-scores were categorized as being within normal limits (WNL), reduced, or elevated, using established cut-off scores. For measures that assess positive traits, a *T*-score WNL was >40, and *T*-scores < 40 were considered clinically “reduced” levels of that positive trait. For measures assessing negative traits, a *T*-score <60 was WNL, and *T*-scores > 60 were considered clinically “elevated” levels of that negative trait. We recorded the frequency at which participants worsened (i.e. WNL at preseason and elevated/reduced at postseason), remained the same, or improved (i.e. elevated/reduced at preseason and WNL at postseason) after the season.

## Results

On all measures, the sample in this study appeared to have similar characteristics as the normative sample, such that the average *T*-scores were approximately 50 (i.e., *M* = 49.8; range = 46.5 to 56.2) with standard deviations of about 10 (i.e., *M* = 10.2; range = 8.1 to 11.8). Table 2 summarizes the descriptive statistics for all seven HRQoL measures at pre and postseason evaluations. At preseason, there were several statistically significant correlations between measures. Higher scores on General Life Satisfaction were associated with greater Emotional Support (*r* = 0.29, *p* = 0.008), and lower symptoms of Psychological Stress (*r* = −0.29, *p* = 0.008) and Depression (*r* = −0.24, *p* = 0.03). Correlations between the PROMIS Physical Stress, Sleep Disturbance, Psychological Stress, and Depression measures were all significant, such that higher scores on any one measure were associated with significantly greater scores on all others (*r* range = 0.33 to 0.69, *p*s < 0.01; see Table 3). Scores on the Peer Relationships measure had a significant but negative association with scores on the Sleep Disturbance (*r = −*0.26, *p* = 0.02) and Depression measures (*r* = −0.28, *p* = 0.01), though it had a significant and positive association with levels of Emotional Support (*r* = 0.26, *p* = 0.02). Finally, there was a significant and negative relationship between Emotional Support and Psychological Stress (*r* = −0.22, *p* < 0.05). The full table of correlations between measures at preseason are presented in Table 3.

**Table 2.** Preseason to postseason within-person comparisons Table 2 long description.

	Preseason		Postseason					
	*M*	SD	*M*	SD	*t*	*p*	*d*	*r*
NIH toolbox – general life satisfaction	47.9	11.0	47.3	10.8	0.46	0.645	0.05	0.50
PROMIS – physical stress	53.7	10.3	56.2	11.7	**−2.15**	**0.035**	**−0.24**	0.52
PROMIS – sleep disturbance	52.6	9.4	52.8	9.4	−0.20	0.840	−0.02	0.63
PROMIS – psychological stress	49.3	10.4	52.3	10.5	**−2.50**	**0.014**	**−0.28**	0.44
PROMIS – depression	47.3	10.7	50.0	11.8	−1.97	0.052	−0.22	0.39
NIH toolbox – emotional support	48.5	9.6	46.6	10.8	1.77	0.081	0.19	0.55
PROMIS – peer relationships	46.6	8.1	46.5	8.8	0.09	0.926	0.01	0.33

*Note*: *N* = 83; *r* = test-retest correlation; NIH = National Institutes of Health; PROMIS = Patient-Reported Outcomes Measurement Information System.

Bold values indicate a significant difference (i.e., *p* < 0.05) in scores from preseason to postseason.

**Table 3.** Inter-test correlations at preseason evaluation Table 3 long description.

	1.	2.	3.	4.	5.	6.
1. NIH toolbox – general life satisfaction	1					
2. PROMIS – physical stress	0.02	1				
3. PROMIS – sleep disturbance	0.03	**0.33**	1			
4. PROMIS – psychological stress	**−0.29**	**0.41**	**0.37**	1		
5. PROMIS – depression	**−0.24**	**0.38**	**0.45**	**0.69**	1	
6. NIH toolbox – emotional support	**0.29**	−0.09	−0.07	**−0.22**	−0.19	1
7. PROMIS – peer relationships	0.17	−0.18	**−0.26**	−0.18	**−0.28**	**0.26**

*Note*: *N* = 83; NIH = National Institutes of Health; PROMIS = Patient-Reported Outcomes Measurement Information System.

Bold values were significant at *p* < 0.05.

At the postseason evaluation, correlations between the PROMIS Physical Stress, Sleep Disturbance, Psychological Stress, and Depression measures remained significant (*r* range = 0.31 to 0.72, *ps* < 0.01; see Table 4). General Life Satisfaction had significant but negative associations with Sleep Disturbance (*r = −*0.22, *p* < 0.01), Psychological Stress (*r* = −0.32, *p* = 0.003), and Depression (*r* = −0.31, *p* = 0.004) but positive associations with Emotional Support (*r* = 0.45, *p* < 0.001) and Peer Relationships (*r* = 0.22, *p* < 0.05). Emotional Support was also negatively associated with Physical Stress, Sleep Disturbance, Psychological Stress, and Depression at postseason (*r* range = −0.27 to −0.41, *ps* < 0.05). The full table of correlations between measures at postseason are presented in Table 4.

**Table 4.** Inter-test correlations at postseason evaluation Table 4 long description.

	1.	2.	3.	4.	5.	6.
1. NIH toolbox – general life satisfaction	1					
2. PROMIS – physical stress	−0.09	1				
3. PROMIS – sleep disturbance	**−0.22**	**0.31**	1			
4. PROMIS – psychological stress	**−0.32**	**0.58**	**0.42**	1		
5. PROMIS – depression	**−0.31**	**0.52**	**0.47**	**0.72**	1	
6. NIH toolbox – emotional support	**0.45**	**−0.27**	**−0.35**	**−0.40**	**−0.41**	1
7. PROMIS – peer relationships	**0.22**	0.00	−0.16	**−0.24**	−0.18	**0.41**

*Note*: *N* = 83; NIH = National Institutes of Health; PROMIS = Patient-Reported Outcomes Measurement Information System.

Bold values were significant at *p* < 0.05.

When comparing preseason to postseason scores using a series of within-persons *t*-tests, there were no statistically significant differences across time points in general Life Satisfaction (*t* = 0.46, *p* = 0.65), Sleep Disturbances (*t* = −0.20, *p* = 0.84), Depression Symptoms (*t* = −1.97, *p* = 0.052), Emotional Support (*t* = 1.77, *p* = 0.08) and Peer Relationships (*t* = 0.09, *p* = 0. 93). Scores significantly increased from pre- to postseason for Physical Stress (*t* = −2.15, *p* = 0.035, *d* = −0.24) and Psychological Stress (*t* = −2.50, *p* = 0.014, *d* = −0.28), although both changes were of a small effect size. Table 2 shows the full comparison of pre- to postseason scores.

We also summarized how participants’ scores on the HRQoL measures may have worsened, remained the same, or improved following a season of football. Overall, most participants showed no change in score classification from pre- to postseason evaluation, and the portion of participants that did not change ranged from 69.9% to 84.3% for all seven measures (see Table 5). Meaning, for the majority of participants, a score that was WNL, reduced (i.e., positive traits), or elevated (i.e., negative traits) at preseason remained so at postseason. For the Physical Stress, Psychological Stress, Depression, Emotional Support, and Peer Relationships measures, more participants worsened (range: 12.0% to 24.1%) than improved (range: 2.4% to 9.6%) following the season. For General Life Satisfaction and Sleep Disturbance, more participants improved (range: 12.0% to 19.3%) than worsened (3.6% to 10.8%).

**Table 5.** Change in score status from pre- to postseason evaluation Table 5 long description.

Outcome measures	*Worsened*		*No change*		*Improved*	
	*n*	%	*n*	%	*n*	%
NIH toolbox – general life satisfaction	9	10.8	58	69.9	16	19.3
PROMIS – physical stress	20	24.1	59	71.1	4	4.8
PROMIS – sleep disturbance	3	3.6	70	84.3	10	12.0
PROMIS – psychological stress	15	18.1	60	72.3	8	9.6
PROMIS – depression	12	14.5	66	79.5	5	6.0
NIH Toolbox – emotional support	15	18.1	66	79.5	2	2.4
PROMIS – peer relationships	10	12.0	65	78.3	8	9.6

*Note*: *N* = 83; NIH = National Institutes of Health; PROMIS = Patient-Reported Outcomes Measurement Information System.

A participant in the worsened category had a normal preseason score followed by a reduced/elevated score at postseason evaluation. A participant in the no change category had pre- and postseason scores that were both within the same normative classification (i.e., within normal limits, reduced/elevated). A participant in the improved category had a reduced/elevated preseason score followed by a WNL score at postseason evaluation.

### Comparison of included and excluded participants

Excluded participants did not have postseason data available; but, as post hoc comparisons, we examined rates of reduced/elevated *T*-scores and sociodemographic characteristics among included and excluded participants to assess for potential differences. There were no meaningful differences in sociodemographic stratifications or rates of health conditions between included and excluded participants (see Table 1). See Table 5 for all HRQoL scores of excluded participants. There were also no meaningful differences in the portion of participants who obtained *T*-scores that were reduced/elevated (see Table 6).

**Table 6.** Rates of reduced (T < 40) or elevated (T > 60) scores at preseason evaluation Table 6 long description.

Outcome measures	*Included participants* (*n* = 83)		*Excluded participants* (*n* = 43)	
	*n*	%	*n*	%
General life satisfaction	23	27.7	13	30.2
Physical stress	22	26.5	14	32.6
Sleep disturbance	19	22.9	13	30.2
Psychological stress	15	18.1	9	20.9
Depression	13	15.7	6	14.0
Emotional support	17	20.5	8	18.6
Peer relationships	18	21.7	7	16.3

*Note:* A *T*-score was considered reduced/elevated if it was at least one SD below or above the mean for positive and negative traits, respectively. For the following scales a *T*-score <40 indicated a reduced score: General Life Satisfaction, Emotional Support, and Peer Relationships. For the following scales a *T*-score >60 indicated an elevated score: Physical Stress, Sleep Disturbance, Psychological Stress, Depression, Emotional Support, and Peer Relationship.

## Discussion

This study examined changes in pediatric HRQoL and well-being measures among a sample of Black or African American high school football players from rural communities across Alabama from preseason to postseason, with the intent of better understanding the effects of high school football participation. The sample’s HRQoL and well-being scores remained WNL before and after the season (within one SD, i.e., *T*-score = ±10, of the population mean of 50). This finding suggests, on average, there is an absence of adverse effects on HRQoL/well-being associated with one season of football participation. However, some variables, specifically physical and psychological stress, slightly increased from preseason to postseason. That said, many other variables (i.e., general life satisfaction, sleep quality, and peer relationships) remained consistent, while emotional support marginally worsened. Overall, this initial effort demonstrates minimal or no change in HRQoL over the course of a single football season among this rural cohort. Findings do not indicate a substantial adverse effect of participating in this team sport on the psychological, behavioral, and social health of participants.

### Sample characteristics

The social, environmental and geographical context of these data used in this analysis has implications for interpreting the results. Previous research has referenced poorer health outcomes for residents of rural communities, and specifically, residents who identify as Black or African American [32]. This may help to explain why the sample’s preseason health history section identified that many athletes reported history of headaches (72.3% included, 74.4% excluded), history of migraines (39.8% included, 44.2% excluded), anxiety and/or depression (36.2% included, 37.2% excluded) before the sport season began [32–34]. While these proportions are on the higher side, they are not excessively high compared to what has been reported in this age group across the general population [35,36]. Despite high incidence of comorbidities that are considered detrimental in terms of HRQoL, this study showed that most athletes self-reported HRQoL values that would be considered typical or healthy at both preseason and postseason.

Researchers have also identified a dose-response relationship between extracurricular sport participation, and both HRQoL and health behaviors, in that youth who engaged in a higher volume of sport activity have better HRQoL than adolescents not as actively engaged in sports [37]. Moreover, extracurricular involvement among underrepresented youth is positively associated with desirable health-related behaviors [38]. Among participants in this study, the majority of football athletes across rural Alabama reported high or good general life satisfaction. At preseason, higher life satisfaction was positively linked to better emotional support, lower psychological stress and lower depression symptoms. These positive correlations remained consistent at postseason also, including better peer relationships and lower sleep disturbances. While examining the results, participating in a team sport could be contributing to the overall normal HRQoL and well-being of all the participants in at least one domain.

Participating in team sports can benefit well-being and promote physical health as individuals move through the lifespan because it provides opportunities for participants to meet or exceed physical activity standards, garnering improved cardiovascular health and lower body fat percentage [39]. From a psychosocial perspective, youth participating in team sport demonstrated increased social cohesion, self-esteem, emotional intelligence, and decreased rates of anxiety and depression [15,39]. Adolescent athletes generally have higher HRQoL than non-athlete adolescents, particularly in emotional functioning [40]. Moreover, the health benefits and behaviors gained through team sport participation as an adolescent can carry into adulthood [15].

### Preseason to postseason assessment of HRQoL scores

Life satisfaction is a subjective measure of fulfillment and quality of one’s life and is a strong indicator of pediatric well-being. Across time points from preseason to postseason, there were no statistically significant differences noted in football athletes’ life satisfaction. Roughly 64% of athletes reported normal or healthy life satisfaction across the football season and 23% of the football athletes reported an improved life satisfaction score at the postseason evaluation. Simultaneously, no changes were noted from preseason to postseason for sleep disturbances, depression symptoms, emotional support, and peer relationships. The majority of football athletes reported normal or improved scores on each of these measures of HRQoL and well-being, demonstrating no adverse effect of football participation on these variables. Although prior research has established some health risks (i.e. concussion, orthopedic injury) associated with football participation [20]. our findings suggest that a season of football participation may have a positive influence on certain domains of well-being for some athletes. For example, 1-in-5 athletes in our sample reported improvements in life satisfaction and 1-in-10 reported improvements in sleep, while the majority reported no change across all HRQoL variables. Team sports like football can provide a sense of community to participants and enhance social health outcomes by providing a dependable social network that is readily available [15]. This could be why we saw that roughly 76% of athletes in this sample reported having positive peer relationships that improved or remained WNL over the duration of the season. Having a sense of community has been shown to mitigate or buffer against risk factors that may lead to adverse HRQoL and well-being in Black and African American communities by reducing social anxiety, facilitating social support, and leveraging health-promotion behaviors that are positively associated with well-being [41,42].

Slight increases were found in physical and psychological stress from preseason to postseason. Physical stress experiences manifest differently from psychological stress experiences and previous studies have explored the minimal clinically important differences for PROMIS measures (i.e. the smallest difference in scores across time that could be deemed important by clinicians) [43]. Our study did not calculate the magnitude of individual change scores across participants, but the overall magnitude across the entire sample was small (2.5 point change on the *T*-score scale), a difference likely of little concern for clinical practice. With that said, it should also not be ignored that some athletes worsened in their stress ratings at postseason. Among athletes with typical physical and psychological stress at preseason, 17.5% had elevated physical stress and 14.3% had elevated psychological stress at postseason. This study only acquired data at the preseason and postseason and did not capture data within the season. For athletes, there are many stressors alongside those that may be associated with football participation, including schoolwork, holidays, and interpersonal relationships with peers and family. It is also possible that the football season coming to an end may have contributed to greater psychological stress because a primary source of social support ended. Additionally, the increase in physical stress may relate to the cumulative toll of the season, resulting in greater bodily pain and headache, or even other musculoskeletal injury that may have occurred during the football season. Reduced HRQoL has been demonstrated in former Division I athletes who played a collision sport particularly for physical domains that include bodily pain and physical functioning [44]. Thus, future research is needed to further identify specific physical and psychological stressors and the timing at which they may cause the most impact to athletes’ HRQoL and well-being.

Trends in football participation have shown a decline across the US and the sport is often criticized for its high degree of contact, leading to concerns among parents and constituents about long-term effects on musculoskeletal health, brain health, and well-being. Few studies discuss or investigate how team sports such as football may be positive for youth development or how team sports like football may support multiple domains of HRQoL and well-being among adolescents. There are well-established health benefits of participating in organized sport as a means of obtaining recommended minutes of moderate-to-vigorous physical activity [45]. In fact, increasing sport participation among children and adolescents is included as an objective in Healthy People 2030 because sport is a popular avenue to accrue these health benefits [46]. Our findings demonstrate that there was not a substantial negative impact of a single season of play across domains of mental, physical, social and emotional HRQoL and well-being in a sample of high school adolescents residing in rural communities.

Football provides opportunities for physical activity and social support through team sport participation for a large number of high school students [39]. Football is also a sport that can facilitate a large number of participants compared to sports such as basketball or baseball where there are limited roster spots. Data from the Aspen Institute 2022 US Report Card demonstrate that sedentary behavior is on the rise among youth whereas organized sport participation, physical fitness, and school-based physical education are all on a consistent decline [18]. Black adolescent students, and those of lower socioeconomic status, also have historically had lower rates of participation in interscholastic or varsity sports compared to their White counterparts and students of higher socioeconomic status [47]. Declines in team sport participation, including football, in rural regions or ethnoracial communities where disparities in sport opportunity are present [47–49] could contribute to reduced physical activity and increased sedentary behavior. This could have secondary adverse effects on HRQoL and physical health (e.g., obesity, chronic disease), which could disproportionately affect Black or African Americans across the lifespan [50].

### Limitations and future directions

The current study reflects a sample of Black or African American male high school football athletes from rural Alabama and provides insight into their psychosocial experience before and after a single season of football participation. This sample may or may not generalize to the entire population of U.S. high school football athletes nor football athletes from other levels of play (e.g., youth, collegiate, professional), but highlights the experience of many communities across the U.S. where football is part of the fabric of high school athletics. The questionnaires administered to participants were based on self-report, without informant report, clinical interviewing, or any physiological measures of the constructs captured (e.g., physical stress). As previously noted, our measures of HRQoL and well-being were administered at the preseason and postseason and not within the season. Further, the measures used did not capture specific factors that may have affected changes from pre-to-postseason (e.g., season record, athletic identity, non-academic/athletic circumstances). As a result, future research may benefit from more in-depth examinations of specific stressors and additional factors that affect HRQoL in adolescents over years of football participation through high school. Future investigation into HRQoL in adolescents could also include additional comparison groups, such as limited contact sport athletes and/or non-athletes. Finally, participant attrition was a limitation as we lost 44 participants from preseason to postseason; those participants were excluded for having incomplete data.

## Conclusion

In conclusion, HRQoL remained WNL and unchanged in the majority of football athletes in this sample over the duration of a single football season. These results show typical levels of HRQoL and psychosocial well-being for the sample both preseason and postseason. Though the risks associated with contact/collision sports, such as concussions, should not be ignored, it is imperative to not prematurely associate football solely with negative consequences. Research has demonstrated numerous positive short- and long-term effects on health due to youth and adolescent team sport participation [39,42,51], and for rural communities where sport equity, resources and accessibility is limited [47–49], football may be the most viable option, unless substantial investment is made to collaborate with communities to discuss alternative sport interests and then create infrastructure (e.g., building new facilities, purchasing equipment) and hire personnel (e.g., coaching, referees) to allow for alternative sporting opportunities. Consequently, there is a need for continued exploration of HRQoL and well-being in adolescent football participants, particularly those originating from less examined and underrepresented rural areas.

## Table 1. Long description

A table comparing health history and socio-demographic characteristics of included and excluded participants in a study of American football players. The table has 23 rows and 6 columns. The columns are labeled Health history characteristics, Included participants (n = 83) n, Included participants (n = 83) %, Excluded participants (n = 43) n, and Excluded participants (n = 43) %. The rows are labeled with various health history characteristics such as Age, Gender, Race, Prior concussion, Number of prior concussions, Undiagnosed concussions, Skipped a year of school, Repeated a year of school, Previous history of: Headaches, Migraines, Motion sickness, Brain Injury/surgery, Seizure/epilepsy, Amblyopia/lazy eye, Vision problems, Anxiety, Depression, ADD/ADHD, Autism/asperger’s, Learning disorders, and Eligible for free/reduced lunch. Each row provides data for included and excluded participants in terms of counts and percentages. For example, the Age row shows that the mean age for included participants is 15.62 with a standard deviation of 1.1, while for excluded participants, the mean age is 15.76 with a standard deviation of 1.4. The Gender row indicates that all included participants are male, while excluded participants are 97.7 percent male and 2.3 percent female. The Race row shows that all included participants are Black, while excluded participants are 20.9 percent White, 72.1 percent Black, 2.3 percent Asian, and 4.7 percent Multiracial. The Prior concussion row shows that 18.1 percent of included participants and 14.0 percent of excluded participants reported a history of concussion. The Number of prior concussions row provides a breakdown of the number of prior concussions reported by participants. The Previous history of row provides data on various health conditions, such as headaches, migraines, motion sickness, brain injury/surgery, seizure/epilepsy, amblyopia/lazy eye, vision problems, anxiety, depression, ADD/ADHD, autism/asperger’s, learning disorders, and eligibility for free/reduced lunch. For example, 72.3 percent of included participants and 74.4 percent of excluded participants reported a history of headaches. 92.8 percent of included participants and 74.4 percent of excluded participants are eligible for free/reduced lunch.

Navigate back to Table 1..

## Table 2. Long description

A table comparing preseason and postseason scores for various health-related quality of life measures. The table has 8 rows and 8 columns. The columns are labeled Preseason M, Preseason SD, Postseason M, Postseason SD, t, p, d, and r. The rows are labeled with different measures: NIH toolbox - general life satisfaction, PROMIS - physical stress, PROMIS - sleep disturbance, PROMIS - psychological stress, PROMIS - depression, NIH toolbox - emotional support, and PROMIS - peer relationships. Each row provides the mean (M), standard deviation (SD), t-value, p-value, d-value, and r-value for the respective measure at preseason and postseason. Notable trends include a significant decrease in PROMIS - physical stress and PROMIS - psychological stress from preseason to postseason, as indicated by the negative t-values and p-values less than 0.05. Other measures show varying degrees of change, with some showing significant correlations between preseason and postseason scores.

Navigate back to Table 2..

## Table 3. Long description

A table showing correlations between various health-related quality of life measures at preseason evaluation. The table has seven rows and seven columns, including row and column headers. The row labels and column headers are the same and include: 1. NIH toolbox - general life satisfaction, 2. PROMIS - physical stress, 3. PROMIS - sleep disturbance, 4. PROMIS - psychological stress, 5. PROMIS - depression, 6. NIH toolbox - emotional support, 7. PROMIS - peer relationships. Row 1: NIH toolbox - general life satisfaction, 1, 0.02, 0.03, -0.29, -0.24, 0.29, 0.17. Row 2: PROMIS - physical stress, 0.02, 1, 0.33, 0.41, 0.38, -0.09, -0.18. Row 3: PROMIS - sleep disturbance, 0.03, 0.33, 1, 0.37, 0.45, -0.07, -0.26. Row 4: PROMIS - psychological stress, -0.29, 0.41, 0.37, 1, 0.69, -0.22, -0.18. Row 5: PROMIS - depression, -0.24, 0.38, 0.45, 0.69, 1, -0.19, -0.28. Row 6: NIH toolbox - emotional support, 0.29, -0.09, -0.07, -0.22, -0.19, 1, 0.26. Row 7: PROMIS - peer relationships, 0.17, -0.18, -0.26, -0.18, -0.28, 0.26, 1.

Navigate back to Table 3..

## Table 4. Long description

A table comparing correlations between various PROMIS measures and general life satisfaction at postseason evaluation. The table has seven rows and six columns. Column headers are labeled as 1, 2, 3, 4, 5, and 6. Row labels are labeled as 1. NIH toolbox – general life satisfaction, 2. PROMIS – physical stress, 3. PROMIS – sleep disturbance, 4. PROMIS – psychological stress, 5. PROMIS – depression, 6. NIH toolbox – emotional support, and 7. PROMIS – peer relationships. Row 1: 1, 0.09, 0.22, 0.32, 0.31, 0.45. Row 2: 0.09, 1, 0.31, 0.58, 0.52, 0.27. Row 3: 0.22, 0.31, 1, 0.42, 0.47, 0.35. Row 4: 0.32, 0.58, 0.42, 1, 0.72, 0.40. Row 5: 0.31, 0.52, 0.47, 0.72, 1, 0.41. Row 6: 0.45, 0.27, 0.35, 0.40, 0.41, 1. Row 7: 0.22, 0.00, 0.16, 0.24, 0.18, 0.41.

Navigate back to Table 4..

## Table 5. Long description

A table comparing changes in scores for various health-related quality of life measures from pre- to postseason evaluation. The table has 7 rows and 6 columns. The columns are labeled as Outcome measures, Worsened (n, %), No change (n, %), and Improved (n, %). The rows are labeled with different outcome measures: NIH toolbox – general life satisfaction, PROMIS – physical stress, PROMIS – sleep disturbance, PROMIS – psychological stress, PROMIS – depression, NIH Toolbox – emotional support, and PROMIS – peer relationships. Each row provides the number and percentage of participants whose scores worsened, showed no change, or improved for each measure. For NIH toolbox – general life satisfaction, 10.8 percent worsened, 69.9 percent showed no change, and 19.3 percent improved. For PROMIS – physical stress, 24.1 percent worsened, 71.1 percent showed no change, and 4.8 percent improved. For PROMIS – sleep disturbance, 3.6 percent worsened, 84.3 percent showed no change, and 12.0 percent improved. For PROMIS – psychological stress, 18.1 percent worsened, 72.3 percent showed no change, and 9.6 percent improved. For PROMIS – depression, 14.5 percent worsened, 79.5 percent showed no change, and 6.0 percent improved. For NIH Toolbox – emotional support, 18.1 percent worsened, 79.5 percent showed no change, and 2.4 percent improved. For PROMIS – peer relationships, 12.0 percent worsened, 78.3 percent showed no change, and 9.6 percent improved.

Navigate back to Table 5..

## Table 6. Long description

A table comparing outcome measures between included and excluded participants. The table has two main columns: Included participants and Excluded participants, each with sub-columns for the number of participants (n) and the percentage (%). The table includes seven outcome measures: General life satisfaction, Physical stress, Sleep disturbance, Psychological stress, Depression, Emotional support, and Peer relationships. Row 1: General life satisfaction, Included participants: n = 23, 27.7 percent, Excluded participants: n = 13, 30.2 percent. Row 2: Physical stress, Included participants: n = 22, 26.5 percent, Excluded participants: n = 14, 32.6 percent. Row 3: Sleep disturbance, Included participants: n = 19, 22.9 percent, Excluded participants: n = 13, 30.2 percent. Row 4: Psychological stress, Included participants: n = 15, 18.1 percent, Excluded participants: n = 9, 20.9 percent. Row 5: Depression, Included participants: n = 13, 15.7 percent, Excluded participants: n = 6, 14.0 percent. Row 6: Emotional support, Included participants: n = 17, 20.5 percent, Excluded participants: n = 8, 18.6 percent. Row 7: Peer relationships, Included participants: n = 18, 21.7 percent, Excluded participants: n = 7, 16.3 percent.

Navigate back to Table 6..
